# Evaluating and volunteering for crowdsourced interventions: Cross-sectional data on COVID-19 safety from a University Survey

**DOI:** 10.1371/journal.pone.0275127

**Published:** 2022-09-29

**Authors:** Suzanne Day, Takhona Grace Hlatshwako, Anna Lloyd, Larry Han, Weiming Tang, Barry Bayus, Joseph D. Tucker

**Affiliations:** 1 Division of Infectious Diseases, Department of Medicine, University of North Carolina at Chapel Hill, Chapel Hill, North Carolina, United States of America; 2 Institute for Global Health and Infectious Diseases, University of North Carolina at Chapel Hill, Chapel Hill, North Carolina, United States of America; 3 Department of Health Policy and Management, Gillings School of Global Public Health, University of North Carolina at Chapel Hill, Chapel Hill, North Carolina, United States of America; 4 Department of Biology, University of North Carolina at Chapel Hill, Chapel Hill, North Carolina, United States of America; 5 Department of Biostatistics, Harvard T.H. Chan School of Public Health, Boston, Massachusetts, United States of America; 6 Social Entrepreneurship to Spur Health (SESH), Guangzhou, China; 7 University of North Carolina at Chapel Hill Project–China, Guangzhou, China; 8 Kenan-Flagler Business School, University of North Carolina at Chapel Hill, Chapel Hill, North Carolina, United States of America; 9 Faculty of Infectious and Tropical Diseases, London School of Hygiene and Tropical Medicine, London, England; Central China Normal University, CHINA

## Abstract

Despite many innovative ideas generated in response to COVID-19, few studies have examined community preferences for these ideas. Our study aimed to determine university community members’ preferences for three novel ideas identified through a crowdsourcing open call at the University of North Carolina (UNC) for making campus safer in the pandemic, as compared to existing (i.e. pre-COVID-19) resources. An online survey was conducted from March 30, 2021 –May 6, 2021. Survey participants included UNC students, staff, faculty, and others. The online survey was distributed using UNC’s mass email listserv and research directory, departmental listservs, and student text groups. Collected data included participant demographics, COVID-19 prevention behaviors, preferences for finalist ideas vs. existing resources in three domains (graduate student supports, campus tours, and online learning), and interest in volunteering with finalist teams. In total 437 survey responses were received from 228 (52%) staff, 119 (27%) students, 78 (18%) faculty, and 12 (3%) others. Most participants were older than age 30 years (309; 71%), women (332, 78%), and white (363, 83.1%). Five participants (1%) were gender minorities, 66 (15%) identified as racial/ethnic minorities, and 46 (10%) had a disability. Most participants preferred the finalist idea for a virtual campus tour of UNC’s lesser-known history compared to the existing campus tour (52.2% vs. 16.0%). For graduate student supports, 41.4% of participants indicated no preference between the finalist idea and existing supports; for online learning resources, the existing resource was preferred compared to the finalist idea (41.6% vs. 30.4%). Most participants agreed that finalists’ ideas would have a positive impact on campus safety during COVID-19 (81.2%, 79.6%, and 79.2% for finalist ideas 1, 2 and 3 respectively). 61 (14.1%) participants indicated interest in volunteering with finalist teams. Together these findings contribute to the development and implementation of community-engaged crowdsourced campus safety interventions during COVID-19.

## Introduction

Throughout the COVID-19 pandemic, much work has been done to address safety and reduce transmission risk on university campuses. Common strategies have included shifting towards online and remote learning [1], student peer support programs [2], and campus-wide testing [3].

To engage university community members in identifying creative solutions for COVID-19 safety, in the summer of 2020 we conducted a crowdsourcing open call at the University of North Carolina at Chapel Hill (UNC) [4]. This open call resulted in several innovative, community-driven solutions [5]. Finalists were identified by a multidisciplinary panel of 14 judges using a rigorous evaluation process to assess submissions for innovation, feasibility, inclusivity, and potential impact. However, the extent to which finalists’ ideas would appeal to the broader university community, including students, faculty, staff, and other stakeholders, was not investigated.

Few studies have examined preferences for strategies to mitigate the impact of COVID-19 university life, with most studies focusing instead on the pandemic’s effect on mental [6–10] and physical health [11], and behaviors for COVID-19 prevention [12]. In addition, there are typically few opportunities for university community members to provide input in formal planning processes, resulting in top-down planning and implementation in which community members’ preferences are not necessarily reflected [13]. For example, when UNC announced plans for Fall 2020 reopening, faculty and campus staff expressed concerns about a lack of transparency and consultation [14]; ultimately, UNC was forced to walk-back campus reopening plans amidst multiple student COVID-19 outbreaks [15]. University staff report few opportunities to participate in institutional decision-making [16], which may be exacerbated by the pandemic. A recent survey by the American Association of University Professors found that nearly a quarter (23.6%) of participants from a stratified random sample of 585 higher education institutions reported a decline in faculty decision-making influence during the pandemic [17].

To address this gap, we conducted an online survey of university community members as an extension of our previous open call research [4]. Study goals were twofold: first, to understand the preferences of university stakeholders for a select number of our open call finalists’ ideas as compared to existing (i.e. pre-COVID-19) campus resources, and second to assess participants’ willingness to volunteer with finalist teams towards implementing their ideas, indicating community enthusiasm for crowdsourced solutions. The purpose of this manuscript is to describe the development, distribution, and results of the survey.

## Materials and methods

### Survey design

The survey was hosted online via Qualtrics, a secure web-based survey system. Our survey was designed to seek input from the full range of university-affiliated stakeholders at UNC Chapel Hill on preferences for strategies to improve campus safety during the COVID-19 pandemic. These strategies were identified through a crowdsourcing open call contest we conducted in the summer of 2020, just prior to the fall semester of the 2020–2021 school year. Methods for this open call have been previously described in detail [4]. In brief, the Carolina Collective open call sought creative ideas from campus stakeholders (including UNC students, staff, faculty, and others within the community) for ways to re-imagine how we might learn, live, and work together safely as a university community during COVID-19. From the open call’s seven finalist submissions, three finalists’ submissions were selected for use in this subsequent survey study based on the need for brevity as well as the ability to contrast these ideas with existing (i.e. pre-COVID-19) resources at UNC Chapel Hill. A description of the selected three finalist ideas as they were presented to survey participants is provided below in Table 1. In brief, these ideas were: 1) an online peer support system for graduate students (submitted by a team of graduate students); 2) a virtual tour of UNC campus focused on the university’s lesser-known history and informed by an anti-racist approach (submitted by a team of undergraduate students); and 3) a program for developing virtual-reality learning experiences (submitted by a team of undergraduate students working with faculty). Each of these three ideas represent important strategies for mitigating the harms of COVID-19 on university campuses. The peer graduate student support system would address students’ mental health and wellbeing amidst a time of heightened anxiety and depression [18]. The virtual tour would ensure that even amidst travel restrictions and social distancing measures, campus visitations could safely continue–a crucial factor in enrolment decisions [19]. Finally, the virtual reality learning system would address the need for improved tools for online learning in the shift to digital classrooms as a means of reducing COVID transmission [20].

**Table 1:** Description of finalist ideas and corresponding existing resources at UNC, as presented to survey participants.

Description of Finalist Ideas	Description of Corresponding Existing Resource
**Finalist Idea 1:** Graduate students are often isolated in their projects, labs, and cohorts due to the individual nature of graduate projects. Our first finalist idea is to provide a digital space where students can interact with other students outside the research setting, creating a healthier and more balanced graduate student life while also fostering interactions between students of different educational backgrounds. The goal of this idea is to foster an environment where peer networking and interactions between graduate students of all levels and backgrounds is encouraged and facilitated. This project would be focused on improving peer-to-peer networking, resource sharing, accessibility, and connecting students to tools for their success.	**Existing Resource 1:** UNC Graduate School provides an extensive resource guide (named C.H.A.R.T) that links students to various opportunities and communities, such as the 600 student organizations that students can join, and other advising and counseling resources.
**Finalist Idea 2:** Our second open call finalist idea is to provide a digital experience to educate various groups about the history of UNC. This idea will use an interactive map to take the viewer around the university, highlighting key places, people, and events, without needing to step foot on campus. Accessible by any computer or smartphone, this educational resource allows accommodations for people who cannot make it to campus. The tour also gives the public an honest and complete picture of the university’s history. Race is inextricably tied to both the past and present of UNC-Chapel Hill, through buildings, events, funding, and much more. For students of color, knowing that their history is uplifted affords them psychological and physical safety on campus.	**Existing Resource 2:** UNC offers the Black and Blue Tour as an in-person group tour through the Visitors Center, only requiring registration in advance. (The Visitors Center is currently closed and no tours are scheduled due to COVID-19).
**Finalist Idea 3:** COVID-19 has made clear the necessity for virtual education options that can simulate or even augment a classroom environment. Although we all hope that the age of COVID-19 will pass soon, our third open call finalist idea seeks to address this necessity by providing teachers and students with the knowledge, resources, and community to create immersive virtual reality/augmented reality (VR/AR) learning experiences. Even when the age of COVID-19 does indeed pass, this VR/AR learning program will continue to offer greater accessibility to those who are otherwise unable to take full advantage of in-person education.	**Existing Resource 3:** UNC utilizes multiple web platforms to facilitate online learning, such as Zoom, Sakai, and Piazza. Furthermore, The Carolina Office for Online Learning (COOL) partners with UNC-Chapel Hill schools and departments to develop and promote high-quality online education programs.

These finalist ideas were contrasted with current resources for UNC graduate students, tours for campus visitors, and online teaching/learning tools, respectively, that existed pre-COVID-19 (see Table 1). The three selected finalist teams provided permission for use of their ideas in the survey. This cross sectional survey study is reported according to the Consensus-Based Checklist for Reporting of Survey Studies (CROSS) [21]. This study was assessed by the institutional review board of the University of North Carolina at Chapel Hill and was determined to be exempt from the requirement for approval, IRB # 21–0266.

Survey questions included demographic information, COVID-19 prevention behaviors, preferences for finalists’ ideas as compared to existing university resources, and willingness to volunteer with finalists’ teams to contribute towards implementation of their ideas at UNC (see S1 File for full wording of the survey). Questions for COVID-19 prevention behaviors included how frequently participants performed certain behaviors (e.g. mask-wearing, physical distancing, hand-washing). Questions to assess participants’ preferences for open call finalists’ ideas compared to existing university resources were comprised of three sections. First, participants were asked about the appeal of the finalist idea, its ability to improve safety at UNC during COVID-19, and willingness to use the finalist idea (or recommend its use, in the case of participants for whom the finalist idea would not be directly relevant–e.g. non-graduate students responding to questions about supports for graduate students). Second, participants were asked to indicate the extent to which a comparable existing resource was appealing to them, as well as willingness to use it (or recommend its use). Third, participants were asked which they preferred: the finalist idea, the current resource, or no preference between the two. Participants were also offered an optional open text box for explaining their choice.

In a separate form de-linked from the previous three survey response sections (to enhance confidentiality), participants were asked if they would be interested in volunteering with any of the finalist teams to help with implementation of their ideas at UNC. If so, they were asked which of the three ideas they would want to volunteer with, and to provide their name and contact information for future volunteering opportunities. All survey participants were also offered the option to provide their name and contact information (email) to be entered in a random draw to win one of four $25 Amazon gift cards. Participants could enter the draw even if they declined interest in volunteering.

### Survey distribution

The online survey was distributed using multiple digital strategies. Primary distribution involved sending a mass informational email via UNC’s Mass Mail system. This is a mass email listserv that can be used to send messages to any UNC email address that has opted into the listserv. Our Mass Mail message invited all UNC-affiliated individuals to participate in the survey, providing a link to the Qualtrics survey form. We supplemented this mass distribution by reaching out directly to 12 UNC departments’ administrative staff with the request to circulate the survey invitation on their departmental listservs. We also leveraged our authorship group’s membership in UNC GroupMe text message groups to distribute the survey link among UNC student groups. Finally, we posted the survey as a research participation opportunity on the Research For Me @ UNC online directory of recruiting studies.

### Data collection and analysis

Survey responses were collected online from March 30, 2021 to May 6, 2021. Survey participants completed electronic informed consent prior to answering the survey. All survey response data collected from participants were compiled using Microsoft Excel and summarized using descriptive statistics, with the exception of text box responses. Since text responses were optional, these data were not formally included for analysis but rather served as supplemental information for interpreting quantitative results.

## Results

### Survey responses

Our UNC MassMail message was received by 8115 UNC email addresses, including 2820 staff, 1774 faculty, 321 undergraduate students, 566 graduate students, and 2634 others (e.g. volunteers, deans and department heads, consultants, visiting scholars, and retirees). Of the 12 UNC departments contacted, three confirmed having sent the survey link to their listservs: communications (700 recipients), economics (115 recipients) and global studies (489 recipients). The survey link was also sent to nine UNC GroupMe groups comprising 3355 recipients, and 17 additional potential participants received the survey link by contacting the study team via the Research For Me @ UNC directory. In total, the link to our survey was delivered 12,791 times to UNC community members’ email addresses/phone numbers. The online survey was completed by 437 participants in total.

### Demographic characteristics

Table 2 presents the demographic information for all 437 survey participants.

**Table 2:** Demographic characteristics of survey participants at UNC, 2021 (N = 437).

Demographic Characteristics	No. (%) of participants
UNC affiliation Undergraduate Student Graduate Student Faculty Staff Other	85 (19.5%) 34 (7.8%) 78 (17.8%) 228 (52.2%) 12 (2.7%)
Gender Man Woman Non-binary Transgender Prefer not to say	92 (21.1%) 332 (76.0%) 4 (0.9%) 1 (0.2%) 8 (1.8%)
Racial or Ethnic category Native American, Alaska Native, Native Hawaiian or other Pacific islander Asian Black or African American White LatinX Two or more racial/ethnic categories Prefer not to say	0 (0.0%) 21 (4.8%) 14 (3.2%) 363 (83.1%) 6 (1.4%) 22 (5.0%) 11 (2.5%)
Age ≤30 years >30 years	128 (29.3%) 309 (70.7%)
Mental/Physical Illness or disability Yes No Prefer not to say	46 (10.5%) 368 (84.2%) 23 (5.3%)

Over half (228, 52%) of survey participants were staff, 27% were students (85 undergraduates and 34 undergraduates, representing 19.5% and 7.8% of participants, respectively), and 17.8% (78 total) were faculty. Twelve (2.7%) participants identified as “other” UNC affiliation (e.g. alumni, volunteers, visiting scholars). Most participants were women (332, 78%), with 1% identifying as transgender or non-binary. Most participants indicated their race/ethnicity as white (363, 83.1%); 9.4% identified as a racial/ethnic minority and 5% identified as more than one race/ethnicity. Participants tended to be older than age 30 years (309, 70.7%) and 10.5% had a physical or mental illness or disability.

### COVID-19 prevention behaviors

**Fig 1:**
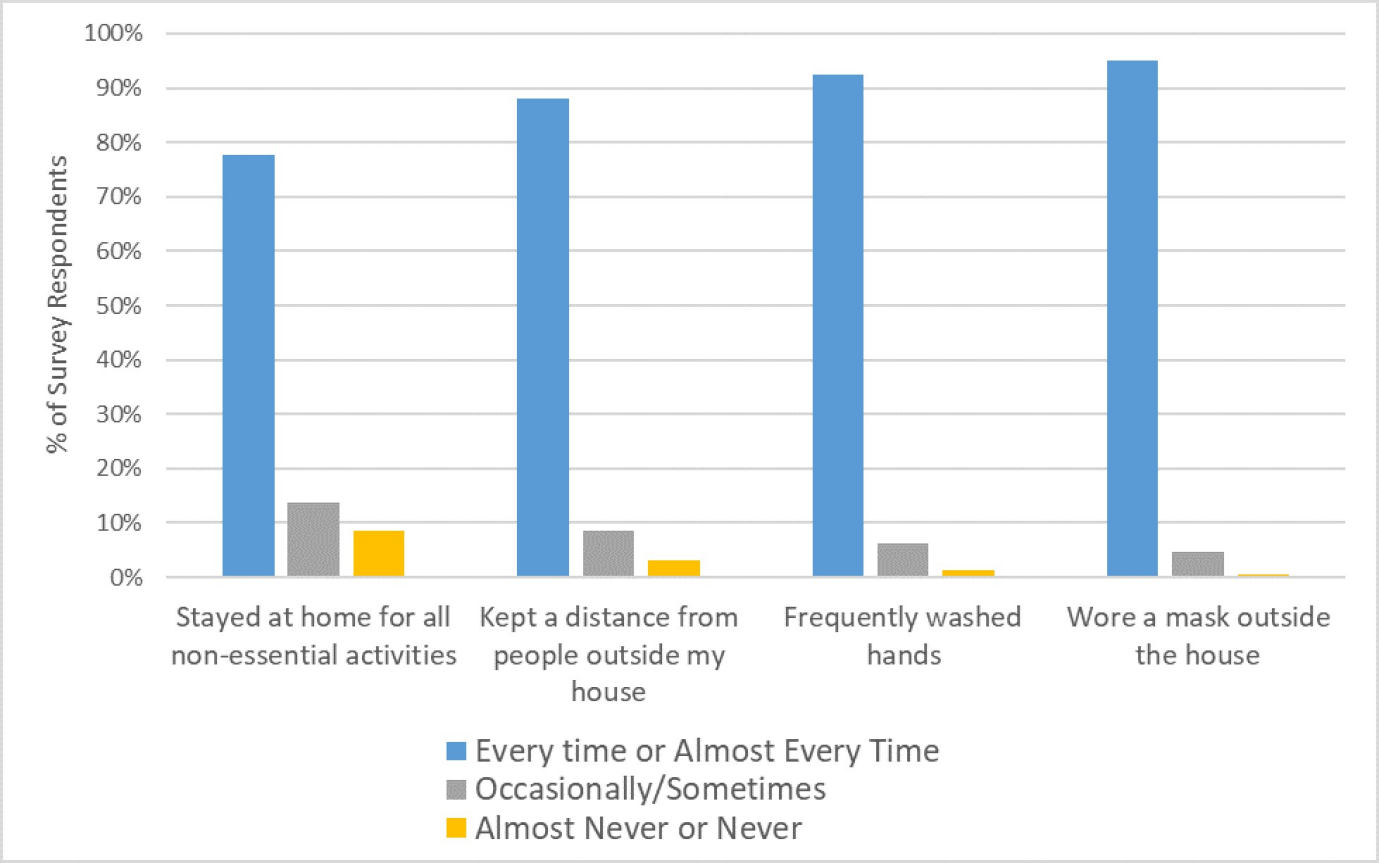
Frequency of COVID-19 prevention behaviors by survey participants in the month prior to survey participation, 2021 (N = 437).

Participants reported a high level of compliance with community safety behaviors (see Fig 1). The majority of participants reported engaging in the following behaviors “every time” or “almost every time” in the past month: stayed home for non-essential activities (340; 77.8%), maintained physical distance from people not in their household (385; 88.1%), washed their hands frequently (404; 92.4%), and wore a mask when leaving the house (415; 94.9%) (see S2 File for additional data on COVID-19 prevention behaviors among participants).

### Preferences for finalist ideas vs. existing resources

Participants’ responses were mixed regarding preferences for the three finalists’ ideas as compared to existing resources (see Table 3). The strongest preference for a finalist idea was in comparing the proposed finalist idea for a virtual campus tour of UNC’s lesser-known history to the existing in-person campus tour, with 228 (52.2%) participants preferring the finalist idea compared to 70 (16.0%) preferring the existing campus tour. In contrast, more participants preferred existing resources for online learning compared to the finalist idea of a program to develop virtual reality learning platforms (41.6% vs. 30.4%, respectively). While more participants preferred the finalist idea for an online peer support network for graduate students compared to current graduate student resources (35.6% vs. 22.8%, respectively), the largest proportion (181; 41.4%) selected “no preference”.

**Table 3:** Survey participants’ preferences for open call finalists’ COVID-19 safety strategies compared to currently existing resources at UNC (N = 437).

Between the current resource at UNC and the new idea proposed by the open call finalists, which do you prefer?	Comparison 1: Supports for graduate students		Comparison 2: Campus tours		Comparison 3: Resources for online learning	
Finalist Idea	156	(35.6%)	228	(52.2%)	133	(30.4%)
Current Resource	100	(22.8%)	70	(16.0%)	182	(41.6%)
No Preference	181	(41.4%)	139	(31.8%)	122	(27.9%)

These patterns in preferences are similarly borne out in comparing responses by UNC affiliation. Table 4 compares preferences for finalist ideas vs. existing resources separately for students (undergraduate and graduate) and employees (faculty and staff). In terms of supports for graduate students, a greater proportion of employees preferred the finalist idea as compared to students (38.2% vs. 26.9%, respectively); however, the greatest proportion of both employees and students indicated no preference in this comparison. In Comparison 2, the finalist idea for campus tours was strongly preferred over existing resources for both student and employee groups, with a greater proportion of employees preferring this idea compared to students (56.9% vs. 42%, respectively). In Comparison 3, the greatest proportion of participants in both the employee and student categories preferred existing resources for online learning, with this preference being stronger in students vs. employees (47.9% vs. 39.9%, respectively).

**Table 4:** Survey participants’ preferences for open call finalists’ COVID-19 safety strategies compared to currently existing resources at UNC, by university affiliation.

Between the current resource at UNC and the new idea proposed by the open call finalists, which do you prefer?		Employees (Faculty & Staff) (N = 306)		Students (Undergrad & Grad) (N = 119)		Other (N = 12)	
Comparison 1: Supports for Graduate Students	Finalist idea	117	38.2%	32	26.9%	7	58.3%
	Current Resource	55	18.0%	42	35.3%	3	25.0%
	No preference	134	43.8%	45	37.8%	2	16.7%
Comparison 2: Campus Tours	Finalist idea	174	56.9%	50	42.0%	4	33.3%
	Current Resource	39	12.7%	28	23.5%	3	25.0%
	No preference	93	30.4%	41	34.5%	5	41.7%
Comparison 3: Resources for Online Learning	Finalist idea	89	29.1%	40	33.6%	4	33.3%
	Current Resource	122	39.9%	57	47.9%	3	25.0%
	No preference	95	31.0%	22	18.5%	5	41.7%

**Fig 2:**
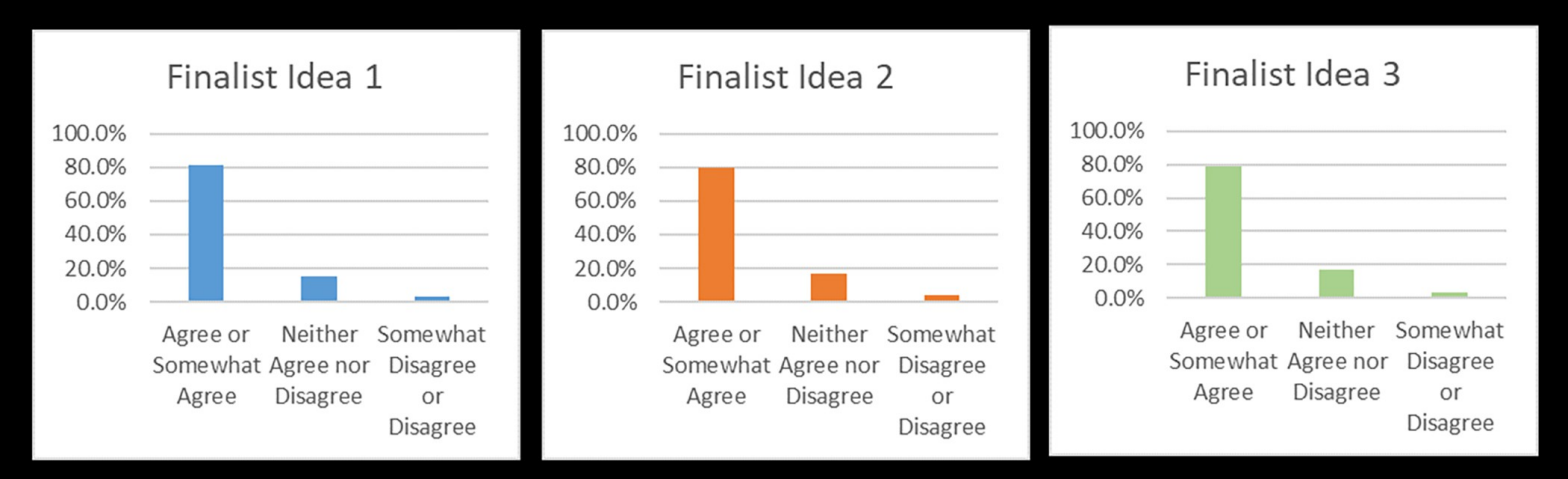
Survey participants’ responses to the statement “In the context of COVID-19 safety measures (i.e. physical distancing), this idea would make UNC a safer place”, by finalist idea (1, 2 and 3) (N = 437).

There was little difference in responses regarding the appeal of finalist and existing resources, as well as likelihood to use/recommend finalist ideas and existing resources (see S3 File). The majority of participants did, however, indicate finalists’ ideas as being beneficial for campus safety, with 355 (81.2%), 348 (79.6%) and 346 (79.2%) of participants agreeing or somewhat agreeing with the statement “In the context of COVID-19 safety measures (i.e. physical distancing), this idea would make UNC a safer place” for finalist ideas 1, 2 and 3, respectively (see Fig 2).

### Interest in volunteering with finalist teams

Of the 437 survey participants, 434 also completed the volunteering portion of the survey (which was de-linked from the previous sections of the survey due to the collection of identifying information). Of these, 61 (14.1%) indicated they would be interested in volunteering with finalist teams to help with implementation. Participants could select one or more team when indicating interest in volunteering; 16 (26.2%) indicated they would be interested in volunteering with the finalist team for implementing an online graduate student support system, 39 (63.9%) selected the virtual campus tour finalist team, and 25 (41.0%) selected the finalist team pursuing a program for developing virtual reality learning.

## Discussion

This survey examined community university members’ preferences for crowdsourced ideas for campus safety during COVID-19 as compared to existing university resources. While the majority of participants agreed that our finalists’ ideas would contribute to making campus safer, there were mixed results in terms of participants’ choice of preference for the finalist ideas vs. comparable existing resources. These findings should be interpreted alongside relevant pandemic- and non-pandemic related contexts.

The finalist idea with the most favorable response from all survey participants (but particularly among staff and faculty) was the virtual tour of UNC campus’ lesser-known history, including the labor of Black enslaved people, systemic racism, and the contributions of UNC students/faculty of color. This result is contextualized by the fact that our survey was conducted at a time of heightened awareness of the need to address widespread systemic racism (as catalyzed by the Black Lives Matter social movement and recognition of racism as a public health crisis) [22], as well as amidst critiques of the university’s failure to acknowledge and address institutional racism at UNC specifically [23]. As such, survey participants’ interest in this finalist idea may not solely be due to its virtual format (compared to an in-person tour), but also due to a desire to address issues of systemic racism on campus, reflecting the broader context of anti-racist action and awareness prompted by recent local and national events.

In contrast, survey participants expressed less enthusiasm for a program that would assist with the creation of virtual reality learning experiences–particularly among students. This result could be attributable to burnout in the shift to online learning and remote working [24], as well as increased “technostress” (e.g. Zoom fatigue) in adapting to new technologies during the pandemic [25]. In addition, our finding that the greatest proportion of all survey participants (41.4%) indicated no preference in the comparison of supports for graduate students is likely attributable to the low rate of survey participation among graduate students, such that participants may have been unable to relate to the question. While we adapted question phrasing for non-graduate student versions of the survey (e.g. replacing “I would make use of this resource” with “I would recommend use of this resource”), more research may be needed to better understand graduate students’ preferences for supportive resources during the pandemic.

Despite mixed results in terms of preferences for finalists’ ideas, that a subset of survey participants indicated willingness to volunteer with one or more finalist teams demonstrates university community members’ enthusiasm for finalists’ ideas, even amidst the substantial competing demands of an ongoing pandemic and the conclusion of the spring 2021 semester (e.g. exams, final assignments). Given the perceived lack of university community involvement in the administration’s planning processes for COVID-19 at UNC [15,26], participants who indicated interest in volunteering may have viewed involvement with finalist teams as an alternative way to contribute to campus safety. Given reports of substantial impacts of the COVID-19 pandemic on mental health and wellbeing among university students, staff and faculty [27], our participants may have viewed volunteering as a means of accomplishing something positive and hopeful amidst the challenges of the pandemic. In addition, volunteering may have been seen as a way to mitigate the limitations on social gathering that were in place at UNC during the time of this survey. Future opportunities should be considered for university community members to volunteer with implementing crowdsourced COVID-19 safety strategies as a way to not only better involve relevant stakeholders, but also as a means for combating pandemic-related isolation.

Our study extends the current literature on COVID-19 considerations among university populations in two ways. First, this study represents a unique contribution to the literature on campus COVID-19 surveys, which have mainly focused on pandemic impact rather than on preferences for how to meet the challenges of the pandemic. Several surveys of university students [6–8] and staff [28] in the U.S. have provided important insights into the toll that the pandemic has had on these populations’ mental health and wellbeing, while other surveys have examined the experiences of students [29] and faculty [30] with adapting to remote learning in the pandemic. The current study adds to the limited literature on community preferences for COVID-19 interventions, as well as the literature on university community members’ willingness to volunteer during the pandemic, which has thus far been limited to studies among medical and nursing students [31–33].

Second, this study contributes to the literature on crowdsourcing as a method for identifying innovative, community-driven responses to the pandemic. While crowdsourcing open calls have been implemented at several universities during COVID-19 [34–37], to date none of the resulting ideas from these open calls have been subsequently evaluated in terms of their appeal to the wider university communities in which they would potentially be implemented. Furthermore, no studies have examined whether the ideas contributed in other COVID-19 related university crowdsourcing open calls would be acceptable to the community of stakeholders among whom the solution would hypothetically be implemented (e.g. students, staff, faculty). Most prior research using crowdsourcing to develop interventions for other health concerns has focused on the effectiveness of crowdsourced interventions rather than stakeholders’ perceptions of crowdsourced ideas–for example, by using randomized trials to assess crowdsourced interventions in behavioral or clinical outcomes [38]. Our study also differs from crowdsourcing projects in which members of the public are invited to vote on contributed ideas as a means of identifying finalists [39,40]. In contrast, our survey involved directly sharing ideas that had already received a top-scoring evaluation from a multidisciplinary panel of judges to determine the extent to which stakeholders would find these ideas appealing, representing a unique approach in obtaining community feedback prior to implementation. While crowdsourcing methodology typically ends with the sharing of proposed solutions and subsequent implementation of ideas, it may be additionally important to gain better insights from the community of affected stakeholders prior to implementation, particularly in settings with heterogeneous stakeholder groups such as university campuses.

This study has four limitations. First, survey distribution was limited to a segment of the UNC community, as there is no means by which all affiliates might be reached via email for non-institutional research. There was additionally potential overlap in our distribution strategies (e.g. persons receiving the survey link via MassMail may have also received it via department listservs), compromising response rate calculation. Our results are thus not necessarily representative of nor generalizable to the entire university community, nor are they reflective of other important stakeholder groups whose views we did not explore due to the logistical challenges of an online survey (e.g. parents, community members surrounding the UNC Chapel Hill campus). In particular, participation among students was low, possibly attributable to survey fatigue given that students had recently received a university-wide COVID-19 survey from UNC administration [41]. Additionally, the low number of responses among racial and ethnic minorities is not reflective of the overall UNC community: in 2020, individuals identifying as racial/ethnic minorities or multi-racial comprised 26.6% of UNC employees (including staff and faculty) and 33.9% of UNC students (including graduate, undergraduate and professional students) [42]. However, only one of the strategies investigated in our survey was exclusively relevant to students (supports for graduate students), while the other two had implications for multiple groups. Additionally, we did receive strong participation from UNC staff and faculty despite the increased work-related stressors of the pandemic [28], suggesting they may be eager to provide input on COVID-19 safety strategies. Second, due to the variability in the number of survey responses obtained per stakeholder group, we are limited to descriptive statistical analyses of respondents’ preferences. Third, given that none of the open call finalists’ ideas had yet been implemented at the time of our survey, participants’ responses were based solely on descriptions of the ideas rather than direct experience with their use. However, investigating participants’ preferences for crowdsourced ideas may be a valuable approach prior to implementation to guide allocation of limited resources. Fourth, by making text responses optional, our survey did not result in sufficient responses for a robust qualitative analysis. Future surveys to assess university community members’ preferences for crowdsourced ideas may consider making text responses required, or offering a multiple-choice menu of explanations to provide further insights.

## Conclusions

Crowdsourced ideas were viewed positively by the university community in terms of their potential to improve campus safety during COVID-19. While crowdsourced ideas were not consistently preferred over existing resources, a subset of participants indicated interest in volunteering with crowdsourcing teams to help with implementation. Providing more opportunities for university community members to get involved with COVID-19 safety strategies (especially community-driven crowdsourced interventions) may be warranted. Our findings contribute new evidence to the rapidly-growing field of research examining university populations during COVID-19, as well as community evaluation of innovative COVID-19 safety strategies identified via crowdsourcing open calls.

## Supporting information

S1 File Full survey text. This supplemental file provides the full text of the online survey (using the Staff Version as an example) in order to provide full information on the survey design.
(DOCX)

S2 File Additional COVID-19 prevention behavior data. This supplemental file presents additional data collected in the survey regarding participants’ comfort with wearing a face mask as a COVID-19 prevention strategy in the month prior to taking the survey (S1 Fig in S2 File) and self-reported intentions to inform others if exhibiting signs of illness during the past month prior to taking the survey (S2 Fig in S2 File).
(DOCX)

S3 File Additional data on survey participants’ preferences for finalist ideas. This supplemental file presents additional data regarding survey participants’ perceptions of the appeal of finalist and existing resources, as well as likelihood to use/recommend finalist ideas and existing resources.
(DOCX)
