# Supplementary material for: Evaluating and volunteering for crowdsourced interventions: Cross-sectional data on COVID-19 safety from a University Survey
Source: PLoS One. 2022 Sep 29;17(9):e0275127. doi: 10.1371/journal.pone.0275127 (PMC9521840; doi:10.1371/journal.pone.0275127)
Supplement: S1 File — This supplemental file provides the full text of the online survey (using the Staff Version as an example) in order to provide full information on the survey design. (DOCX) [file pone.0275127.s001.docx]

Supporting Information 1: Full Survey Text

Carolina Collective Digital Survey – Staff Version

| **Purpose of the Survey** | | |
| --- | --- | --- |
| The purpose of this survey is to evaluate the preferences of UNC staff regarding ideas identified as finalist submissions for the [Carolina Collective open call](https://carolinacollective.web.unc.edu/) – a crowdsourcing contest that was launched in the summer of 2020 to identify creative ways of enhancing safety and well-being at UNC during the COVID-19 pandemic. We’d also like to know how well you think these ideas respond to the challenges faced by UNC due to the COVID-19 pandemic. | | |
| **Description of the Survey** | | |
| This survey has four sections (Socio-demographics, COVID-19 safety, Preferences, and Volunteering) and will take approximately 20 minutes to complete. | | |
|  | **Question** | **Response** |
| **Section 1:**  **Socio-demographics** | 1.What kind of staff position do you hold at UNC? | [Pulldown menu:]  Accounting/Finance/Auditing  Academic Administration  Administrative/Clerical support  Athletics  Development & Fundraising  Engineering & Architecture  Environment, Health & Safety  Facilities & Skilled Trades  Health professional (physician, nurse, other)  Housekeeping & General Services  Human Resources  Information Technology  Lab Management & Services  Libraries  Parking & Transportation  Police/Security  Postdoctoral Scholar  Public Relations/Communications/Marketing  Research Professionals  Student Services  Other |
|  | 2. What is your employment status?  (Please select all that apply) | 1. Full time  2. Part-time  3. Permanent  4. Temporary |
|  | 3.Which of the following options best describes your gender? | 1. Man 2. Woman 3. Non-binary 4. Transgender 5. A gender identity not listed here (Specify) 6. Prefer not to say |
|  | 4.Which of the following options best describes your racial or ethnic category? (Check all that apply) | 1. Native American or Alaska Native 2. Native Hawaiian or other Pacific Islander 3. Asian 4. Black or African American 5. White 6. Latinx 7. A category not listed here (Specify) 8. Prefer not to say |
|  | 5. What is your age? (in years) | (numeric) |
|  | 6. Do you currently have any mental or physical illness or disability that affects you in your everyday life? By affecting your life, we mean limiting your usual activities in any way. | 1. Yes 2. No 3. Prefer not to say |
|  | 7. Prior to this survey, had you heard about the Carolina Collective open call, or participated in submitting an idea to the open call? (select all that apply) | 1. Yes, I had heard of the open call prior to this survey, but I did not submit an idea.  2. Yes, I had heard of the open call prior to this survey, and I submitted an idea either as an individual or as part of a team.  3. No, I have never heard of the Collective open call. |
| **Section 2: COVID-19**  **Safety** | 1.To what extent do the following statements describe your behavior in the past month as a result of COVID-19.  Response options for all questions except 4:  1 – Never  2 – Almost never  3 – Occasionally/Sometimes  4 – Almost every time  5 – Every time  Response options for 4:  1 – Strongly disagree  2 – Disagree  3 – Neither agree or disagree  4 – Agree  5 – Strongly agree | 1. I stayed at home with the exception of essential outings (e.g. grocery shopping, medical appointments). 2. I did not attend social gatherings. 3. I kept a distance of at least 6 feet (2 meters) from people not in my household. 4. If I exhibited symptoms of sickness, I would have immediately informed the people around me. 5. I washed my hands frequently. 6. I wore a mask whenever I left the house. 7. I wore gloves when I left the house. |
|  | 2.How do you feel about wearing a face mask? | 1= Extremely comfortable (no problems whatsoever)  2= Very comfortable (very few problems wearing a face mask)  3= Comfortable (some problems/discomfort)  4= Not very comfortable (had problems/discomforts much of the time)  5= Extremely uncomfortable (had problems/discomforts most to all of the time) |
| **Section 3: Preferences Preamble** | The next section of the survey presents a selection of finalist ideas from the Carolina Collective open call. The Carolina Collective open call was a crowdsourcing contest that was held in the summer of 2020 to obtain ideas from the UNC community about how to enhance safety and wellbeing at UNC in the fall semester. In the following section, you will be asked to read a brief summary of three finalists’ ideas and provide your opinions on them. You will also be presented with information on existing resources and programs at UNC, and asked to provide your opinions on them as compared to the crowdsourced ideas.  Please click to proceed to the next survey section. | |
| **Open Call Finalist 1** | Please read the following summary of our first finalist idea, and answer the questions below:  Graduate students are often isolated in their projects, labs, and cohorts due to the individual nature of graduate projects. Our first finalist idea is to provide a digital space where students can interact with other students outside the research setting, creating a healthier and more balanced graduate student life while also fostering interactions between students of different educational backgrounds. The goal of this idea is to foster an environment where peer networking and interactions between graduate students of all levels and backgrounds is encouraged and facilitated. This project would be focused on improving peer-to-peer networking, resource sharing, accessibility, and connecting students to tools for their success. | |
| Response to finalist idea | How much do you agree with the following statements: | |
|  | 1. This idea is appealing to me. | 1. Agree 2. Somewhat agree 3. Neither agree or disagree 4. Somewhat disagree 5. Disagree |
|  | 2. In the context of COVID-19 safety measures (i.e. physical distancing), this idea would make UNC a safer place | 1. Agree 2. Somewhat agree 3. Neither agree or disagree 4. Somewhat disagree 5. Disagree |
|  | 3. If this idea were implemented at UNC, I would encourage UNC graduate students to make use of it. | 1. Agree 2. Somewhat agree 3. Neither agree or disagree 4. Somewhat disagree 5. Disagree |
|  | 4.Is there any additional feedback or concerns you have about implementing this proposed idea at UNC? | (open text box; optional) |
| Response to current resources | Please take a moment to read about the current resource available for graduate students at UNC, and answer the questions below:  UNC Graduate School provides an extensive resource guide (named C.H.A.R.T) that links students to various opportunities and communities, such as the 600 student organizations that students can join, and other advising and counseling resources. | |
|  | How much do you agree with the following statements: | |
|  | 1. This current resource is appealing to me | 1. Agree  2. Somewhat agree  3. Neither agree or disagree  4. Somewhat disagree  5. Disagree |
|  | 2. I would encourage graduate students to make use of this resource at UNC (or have recommended it to graduate students in the past) | 1. Agree 2. Somewhat agree 3. Neither agree or disagree 4. Somewhat disagree 5. Disagree |
| Preference for current resource vs. finalist idea | Between the current resource at UNC and the new idea proposed by the open call finalists, which do prefer as a resource for UNC graduate students? (Please select one) | |
|  | 1. Current resource: A resource guide (named C.H.A.R.T) that links graduate students to various opportunities and communities, including student organizations and other advising and counseling resources.  2. Finalist idea: Provide a digital space where graduate students can interact with other students outside the research setting, fostering interactions between students of different educational backgrounds.  3. I do not have a preference for one or the other. | |
|  | [Optional]  IF answered 1: Can you explain in a few words why you prefer the current resource? [open text box]  IF answered 2: Can you explain in a few words why you prefer the finalist idea? [open text box]  IF answered 3: Can you explain in a few words why you do not have a preference for one or the other? | |
| **Open Call Finalist 2** | Please take a moment to read about our second open call finalist, and respond to the questions below:  Our second open call finalist idea is to provide a digital experience to educate various groups about the history of UNC. This idea will use an interactive map to take the viewer around the university, highlighting key places, people, and events, without needing to step foot on campus. Accessible by any computer or smartphone, this educational resource allows accommodations for people who cannot make it to campus. The tour also gives the public an honest and complete picture of the university’s history. Race is inextricably tied to both the past and present of UNC-Chapel Hill, through buildings, events, funding, and much more. For students of color, knowing that their history is uplifted affords them psychological and physical safety on campus. | |
| Response to finalist idea | How much do you agree with the following statements: | |
|  | 1.This idea is appealing to me | 1. Agree 2. Somewhat agree 3. Neither agree or disagree 4. Somewhat disagree 5. Disagree |
|  | 2. In the context of COVID-19 safety measures (i.e. physical distancing), this idea would make UNC a safer place | 1. Agree 2. Somewhat agree 3. Neither agree or disagree 4. Somewhat disagree 5. Disagree |
|  | 3. If this intervention were implemented at UNC, I would participate/make use of it. | 1. Agree 2. Somewhat agree 3. Neither agree or disagree 4. Somewhat disagree 5. Disagree |
|  | 4.Is there any additional feedback or concerns you have about implementing this proposed idea at UNC? | (open text box; optional) |
| Response to current resource | Please take a moment to read about the current resource available for touring campus and learning about UNC history, and answer the questions below:  UNC offers the Black and Blue Tour as an in-person group tour through the Visitors Center, only requiring registration in advance. (The Visitors Center is currently closed and no tours are scheduled due to COVID-19). | |
|  | How much do you agree with the following statements: | |
|  | 1.This current resource is appealing to me | 1. Agree  2. Somewhat agree  3. Neither agree or disagree  4. Somewhat disagree  5. Disagree |
|  | 2. I would make use of (or already use) this resource at UNC | 1. Agree  2. Somewhat agree  3. Neither agree or disagree  4. Somewhat disagree  5. Disagree |
| Preference for current resource vs. finalist idea | Between the current resource at UNC and the new idea proposed by the open call finalists, which do you prefer? (Please select one) | |
|  | 1. Current resource: the in-person Black and Blue group tour  2. Finalist idea: Virtual tour of UNC’s lesser-known history.  3. I do not have a preference for one or the other. | |
|  | [Optional]  IF answered 1: Can you explain in a few words why you prefer the current resource? [open text box]  IF answered 2: Can you explain in a few words why you prefer the finalist idea? [open text box]  IF answered 3: Can you explain in a few words why you do not have a preference for one or the other? | |
| **Open Call Finalist 3** | Please take a moment to read about our third open call finalist, and answer the questions below:  COVID-19 has made clear the necessity for virtual education options that can simulate or even augment a classroom environment. Although we all hope that the age of COVID-19 will pass soon, our third open call finalist idea seeks to address this necessity by providing teachers and students with the knowledge, resources, and community to create immersive virtual reality/augmented reality (VR/AR) learning experiences. Even when the age of COVID-19 does indeed pass, this VR/AR learning program will continue to offer greater accessibility to those who are otherwise unable to take full advantage of in-person education. | |
|  | How much do you agree with the following statements: | |
|  | 1. This idea is appealing to me | 1. Agree  2. Somewhat agree  3. Neither agree or disagree  4. Somewhat disagree  5. Disagree |
|  | 2. In the context of COVID-19 safety measures (i.e. physical distancing), this idea would make UNC a safer place | 1. Agree 2. Somewhat agree 3. Neither agree or disagree 4. Somewhat disagree 5. Disagree |
|  | 3. If this idea were implemented at UNC, I would encourage UNC students and instructors to participate/make use of it. | 1. Agree 2. Somewhat agree 3. Neither agree or disagree 4. Somewhat disagree 5. Disagree |
|  | 4.Is there any additional feedback or concerns you have about implementing this proposed idea at UNC? | (open text box; optional) |
| Response to current resource | Please take a moment to read about the current resource available for online learning at UNC, and answer the questions below:  UNC utilizes multiple web platforms to facilitate online learning, such as Zoom, Sakai, and Piazza. Furthermore, The Carolina Office for Online Learning (COOL) partners with UNC-Chapel Hill schools and departments to develop and promote high-quality online education programs. | |
|  | How much do you agree with the following statements: | |
|  | 1. This current resource is appealing to me. | 1. Agree  2. Somewhat agree  3. Neither agree or disagree  4. Somewhat disagree  5. Disagree |
|  | 2. I would make use of this resource at UNC (or have used it in the past) | 1. Agree  2. Somewhat agree  3. Neither agree or disagree  4. Somewhat disagree  5. Disagree |
| Preference for current resource vs. finalist idea | Between the current resource at UNC and the new idea proposed by the open call finalists, which do you prefer? (Please select one) | |
|  | 1. Current resource: existing online learning platforms (Zoom, Sakai, Piazza).  2. Finalist idea: program to support teachers and students in creating immersive virtual reality/augmented reality (VR/AR) learning experiences.  3. I do not have a preference for one or the other. | |
|  | [Optional]  IF answered 1: Can you explain in a few words why you prefer the current resource? [open text box]  IF answered 2: Can you explain in a few words why you prefer the finalist idea? [open text box]  IF answered 3: Can you explain in a few words why you do not have a preference for one or the other? | |
| **Section 4: Volunteering** | All of our finalist teams are interested in moving forward with implementing their proposed ideas at UNC. Would you be interested in volunteering with any of the finalists’ projects described above? | 1. Yes 2. No |
| **DE-LINKED SUBSECTION: collection of personal identifiers** | If yes: which finalist team? (Choose all that apply) | 1. Finalist team 1: digital space for graduate student peer support  2. Finalist team 2: Virtual campus tours  3. Finalist team 3: Program to support the creation of virtual learning experiences |
|  | Please tell us your level of preferred volunteering commitment | 1. I would be interested in helping out a little (small time commitment)  2. I would be interested in helping out a lot (large time commitment)  3. I am not sure how much time I could commit, but I am still interested in volunteering |
|  | If yes: Would you like to provide your name and email address so that we can contact you with future volunteering opportunities with this team (or teams)? | 1. Yes  2. No |
|  | If yes: Please provide your name and email address: | [Open text box: Name]  [Open text box: Email] |
| **Section 5: Draw participation** | 1. Would you like to provide your name and contact information to be entered in a drawing based on chance in which each participant will have equal odds of receiving one of four $25 Amazon gift cards? | 1.Yes  2.No |
| **DE-LINKED SUBSECTION: collection of personal identifiers** | IF YES: Please provide your name and email address to enter the draw. | [Name]  [Email] |
