# Supplementary material for: Evaluating and volunteering for crowdsourced interventions: Cross-sectional data on COVID-19 safety from a University Survey
Source: PLoS One. 2022 Sep 29;17(9):e0275127. doi: 10.1371/journal.pone.0275127 (PMC9521840; doi:10.1371/journal.pone.0275127)
Supplement: S2 File — This supplemental file presents additional data collected in the survey regarding participants’ comfort with wearing a face mask as a COVID-19 prevention strategy in the month prior to taking the survey (S1 Fig in S2 File) and self-reported intentions to inform others if exhibiting signs of illness during the past month prior to taking the survey (S2 Fig in S2 File). (DOCX) [file pone.0275127.s002.docx]

Supporting Information 2: Additional COVID-19 Prevention Behavior Data

**Supplemental Figure 1: Comfort level of survey participants with wearing a face mask as a COVID-19 prevention strategy in the month prior to taking the survey (N=437).**

**Supplemental Figure 2: Survey participants’ self-reported intentions to inform others if exhibiting signs of illness during the past month prior to taking the survey (N=437).**
