# Supplementary material for: Evaluating and volunteering for crowdsourced interventions: Cross-sectional data on COVID-19 safety from a University Survey
Source: PLoS One. 2022 Sep 29;17(9):e0275127. doi: 10.1371/journal.pone.0275127 (PMC9521840; doi:10.1371/journal.pone.0275127)
Supplement: S3 File — This supplemental file presents additional data regarding survey participants’ perceptions of the appeal of finalist and existing resources, as well as likelihood to use/recommend finalist ideas and existing resources. (DOCX) [file pone.0275127.s003.docx]

Supporting Information 3: Additional Data on Survey Participants’ Preferences for Finalist Ideas

**Supplemental Table 1: Comparison of survey participants’ preferences for Finalist Idea 1 vs. existing resource (supports for graduate students) (N=437)**

| COMPARISON 1:  Supports for grad students | | Agree | | Somewhat Agree | | Neither Agree nor Disagree | | Somewhat Disagree | | Disagree | |
| --- | --- | --- | --- | --- | --- | --- | --- | --- | --- | --- | --- |
| Finalist Idea | This idea is appealing to me | 171 | (39%) | 119 | (27%) | 114 | (26%) | 23 | (5%) | 10 | (2%) |
|  | If this idea were implemented at UNC, I would participate/make use of it. | 217 | (50%) | 93 | (21%) | 105 | (24%) | 16 | (4%) | 6 | (1%) |
| Current Resource | This current resource is appealing to me. | 165 | (38%) | 105 | (24%) | 133 | (30%) | 23 | (5%) | 11 | (3%) |
|  | I would make use of (or already use) this resource at UNC. | 212 | (49%) | 108 | (25%) | 100 | (23%) | 10 | (2%) | 7 | (2%) |

**Supplemental Table 2: Comparison of survey participants’ preferences for Finalist Idea 2 vs. existing resource (campus tours) (N=437)**

| COMPARISON 2:  Campus tours | | Agree | | Somewhat Agree | | Neither Agree nor Disagree | | Somewhat Disagree | | Disagree | |
| --- | --- | --- | --- | --- | --- | --- | --- | --- | --- | --- | --- |
| Finalist Idea | This idea is appealing to me | 259 | (59%) | 108 | (25%) | 45 | (10%) | 16 | (4%) | 9 | (2%) |
|  | If this idea were implemented at UNC, I would participate/make use of it. | 195 | (45%) | 115 | (26%) | 77 | (18%) | 26 | (6%) | 24 | (5%) |
| Current Resource | This current resource is appealing to me. | 140 | (32%) | 120 | (27%) | 112 | (26%) | 38 | (9%) | 27 | (6%) |
|  | I would make use of (or already use) this resource at UNC. | 119 | (27%) | 84 | (19%) | 113 | (26%) | 65 | (15%) | 56 | (13%) |

**Supplemental Table 3: Comparison of survey participants’ preferences for Finalist Idea 3 vs. existing resource (resources for online learning) (N=437)**

| COMPARISON 3:  Resources for online learning | | Agree | | Somewhat Agree | | Neither Agree nor Disagree | | Somewhat Disagree | | Disagree | |
| --- | --- | --- | --- | --- | --- | --- | --- | --- | --- | --- | --- |
| Finalist Idea | This idea is appealing to me | 177 | (41%) | 120 | (27%) | 66 | (15%) | 41 | (9%) | 33 | (8%) |
|  | If this idea were implemented at UNC, I would participate/make use of it. | 162 | (37%) | 99 | (23%) | 98 | (22%) | 32 | (7%) | 46 | (11%) |
| Current Resource | This current resource is appealing to me. | 199 | (46%) | 139 | (32%) | 76 | (17%) | 14 | (3%) | 9 | (2%) |
|  | I would make use of (or already use) this resource at UNC. | 261 | (60%) | 117 | (27%) | 50 | (11%) | 4 | (1%) | 5 | (1%) |

**Supplemental Table 4: Survey participants’ preferences for Finalist Idea 1 vs. current resource (graduate student supports), by UNC affiliation (N=437).**

| Between the current resource at UNC and the new idea proposed by the open call finalists, which do you prefer? | Faculty (N=78) | | Undergrad (N=85) | | Grad (N=34) | | Staff (N=228) | | Other (N=12) | |
| --- | --- | --- | --- | --- | --- | --- | --- | --- | --- | --- |
| Finalist idea | 27 | (34.6%) | 23 | (27.1%) | 9 | (26.5%) | 90 | (39.5%) | 7 | (58.3%) |
| Current Resource | 17 | (21.8%) | 33 | (38.8%) | 9 | (26.5%) | 38 | (16.7%) | 3 | (25.0%) |
| No preference | 34 | (43.6%) | 29 | (34.1%) | 16 | (47.1%) | 100 | (43.9%) | 2 | (16.7%) |

**Supplemental Table 5: Survey participants’ preferences for Finalist Idea 2 vs. current resource (campus tours), by UNC affiliation (N=437).**

| Between the current resource at UNC and the new idea proposed by the open call finalists, which do you prefer? | Faculty (N=78) | | Undergrad (N=85) | | Grad (N=34) | | Staff (N=228) | | Other (N=12) | |
| --- | --- | --- | --- | --- | --- | --- | --- | --- | --- | --- |
| Finalist idea | 36 | (46.2%) | 41 | (48.2%) | 9 | (26.5%) | 138 | (60.5%) | 4 | (33.3%) |
| Current Resource | 12 | (15.4%) | 20 | (23.5%) | 8 | (23.5%) | 27 | (11.8%) | 3 | (25.0%) |
| No preference | 30 | (38.5%) | 24 | (28.2%) | 17 | (50.0%) | 63 | (27.6%) | 5 | (41.7%) |

**Supplemental Table 6: Survey participants’ preferences for Finalist Idea 3 vs. current resource (resources for online learning), by UNC affiliation (N=437).**

| Between the current resource at UNC and the new idea proposed by the open call finalists, which do you prefer? | Faculty (N=78) | | Undergrad (N=85) | | Grad (N=34) | | Staff (N=228) | | Other (N=12) | |
| --- | --- | --- | --- | --- | --- | --- | --- | --- | --- | --- |
| Finalist idea | 18 | (23.1%) | 31 | (36.5%) | 9 | (26.5%) | 71 | (31.1%) | 4 | (33.3%) |
| Current Resource | 42 | (53.8%) | 38 | (44.7%) | 19 | (55.9%) | 80 | (35.1%) | 3 | (25.0%) |
| No preference | 18 | (23.1%) | 16 | (18.8%) | 6 | (17.6%) | 77 | (33.8%) | 5 | (41.7%) |
